# Lessons learned from inadequate implementation planning of team-based chronic disease management: implementation evaluation

**DOI:** 10.1186/s12913-021-06100-4

**Published:** 2021-02-12

**Authors:** Shannon L. Sibbald, Rachelle Van Asseldonk, Peiwen L. Cao, Benson Law

**Affiliations:** 1 Faculty of Health Sciences, University of Western Ontario, London, Canada; 2 Department of Family Medicine, Schulich School of Medicine and Dentistry, University of Western Ontario, London, Canada

**Keywords:** Implementation study, COPD, Chronic disease, Change management, Evidence-based practice, Facilitation, Implementation research into practice, Failure

## Abstract

**Background:**

This study was a retrospective evaluation of an unsuccessfully implemented team-based, chronic disease management program, with an aim to understand more about implementation barriers. The program, the Chronic Disease Management Initiative (CDMI) was a new collaborative model of care for patients with COPD. It utilized customized health information and interactive tools, mainly smartphones, for ongoing disease management. The program’s goal was to demonstrate that integrated team-based models of care could improve patient care, as well as reduce readmission rates and emergency department visits. The program planning for CDMI began in 2017, followed by the implementation and evaluation period in 2018. After a 10-month implementation period, the program was unable to enroll a sufficient number of patients to examine if there was an improvement in patient outcomes.

**Methods:**

A retrospective case-study design using multiple data sources was used to gather feedback from participants involved in CDMI. Data collection occurred throughout planning and implementation and continued into early 2019. Semi-structured interviews were conducted, and transcripts were analyzed using NVivo 10 software. This was followed by content analysis.

**Results:**

Analysis revealed four key themes as barriers to CDMI’s implementation: 1) lack of a needs assessment with key stakeholders; 2) lack of buy-in from medical staff; 3) inadequate patient engagement and; 4) contextual barriers. Planners did not conduct a proper needs assessment, nor include patients in the study design. In addition, there was insufficient consideration for how CDMI should be integrated into the usual COPD care plan, leading to confusion in roles and responsibilities. Poor communication between the implementation team and healthcare providers implementing the program, led to a lack of buy-in and engagement.

**Conclusion:**

The key themes resonate with what is already known in the literature. This study supports the importance of using a theoretically grounded plan for implementation. Using a model only in the planning stages is insufficient even when an intervention is based on evidence to support higher quality care. It is imperative to consider practical and contextual factors of program implementation and their interactions. By detailing the ‘failed implementation’ of this intervention, we hope to share important lessons about the need to plan implementation processes early in program planning.

**Supplementary Information:**

The online version contains supplementary material available at 10.1186/s12913-021-06100-4.

## Background

The literature has demonstrated many complex and interconnected factors that can determine implementation success. These include supportive leadership, an enabling organizational culture, patients’ perceptions on the intervention, amongst others [1–3]. It is also acknowledged that these factors interact with one another, although little is known about exactly how context might shift the balance of interconnectivity. The Consolidated Framework for Implementation Research (CFIR) specifically identifies the implementation setting as an important factor, alongside the interplay between individual stakeholders and facilitators [4]. However, more guidance on this interplay is needed. Implementation science is focused on improving the successful uptake of evidence-based methodologies to improve health care quality and overall effectiveness [5]. Evaluating unsuccessful implementations can provide lessons that can be applied prospectively to improve the likelihood of intervention success [6]. However, details of unsuccessful implementations are often under-reported, leaving notable gaps in the literature [7], negatively impacting the quality of care that patients receive, as well as contributing to increased costs and provider workload [8]. Motivated by this gap, we conducted a retrospective evaluation on an unsuccessfully implemented chronic disease management program.

The Chronic Disease Management Initiative (CDMI) program was guided by a chronic disease management model proposed by Bodenheimer and colleagues [9] with six elements: linkage with community resources, buy-in by health care organizations, structured practice teams for chronic care management, self-management support, decision support and clinical information systems that ensured reminders and feedback about patient progress.

CDMI was designed to support patients living with chronic obstructive pulmonary disease (COPD) through an interactive, mobile-based platform delivered in a primary care setting. Participants were given a smartphone for receiving targeted messages from their healthcare providers. As part of the program, patients and providers received a brief training on the use of the device during regularly scheduled visits. Text messages were initiated by a program navigator at least twice a week to reinforce health teaching and monitoring activities. Previous research has shown that some patients prefer text message communication to in-person or phone conversations because it can diminish feelings of embarrassment related to health issues [10]. Another study identified that being able to contact health care providers with a mobile device was like having a “permanently outstretched hand”, even when health care services are not being accessed [11].

After a 10-month implementation period, the CDMI program was unable to enroll a sufficient number of patients to realize any improvement in patient outcomes. As such, researchers were unable to determine whether the smart phone technology, if implemented properly, could actually work to improve communication between patient and provider and, in turn, improve patient outcomes.

The contributions of this study to the implementation science literature include: 1) providing experiential knowledge to guide future health interventions in planning for real world challenges they may encounter, especially on the individual or organizational level; 2) highlighting the importance of involving participants from the outset of planning as it can improve the likelihood of a successful implementation and guide the intervention to fruition; and 3) bridging the gap in the literature where unsuccessful implementations are underreported.

## Methods

Our research was guided by the Consolidated Framework for Implementation Research (CFIR) which helped to identify critical constructs in the implementation process [12]. CDMI’s implementation team (*n* = 11) was made up of researchers (*n* = 2), family physicians (*n =* 2), a respirologist (*n =* 1), nurses (*n =* 2), psychiatrists (*n =* 2), a cardiologist (*n =* 1), and a research coordinator (*n =* 1).

The 10-month intervention period was led by the research coordinator and executed by a subset of the research team. After the unsuccessful implementation, all eleven team members were invited to take part in a semi-structured interview. Four informants participated in-person (36.36% response rate), one participated via email; one explicitly chose not to partake since they believed that they were not integral to CDMI’s implementation; the others absent did not cite a reason for their lack of participation.

Participants were asked eight open-ended questions about their view on the project’s development and implementation, their involvement, team perceptions, as well as areas of improvement (See Additional file 1). Questions were developed to explore the constructs in CFIR [12]. The interviews were transcribed verbatim; NVivo10 was used to support analysis.

The interview data was coded by two researchers (SLS, PC) using a conventional content analysis [13]. The codes and themes were reviewed and refined by the entire research team. Analysis began with an initial read-through of the transcripts to identify significant and relevant content. This process was repeated until the transcripts were fully coded and all relevant content was marked. Data analysis was augmented by looking at related implementation documents such as meeting minutes, project proposals, and ethics documents. Codes were combined into themes and meaningful patterns in the data were examined in relation to all data sources [14]. The STARi checklist by Pinnock and colleagues [15] was used to ensure transparent research reporting (See Additional file 2).

## Results

Four themes were identified from the responses as key barriers to CDMI’s implementation: 1) lack of needs assessments and engagement with key stakeholders; 2) lack of buy-in from medical staff; 3) inadequate patient engagement; and 4) contextual barriers. These themes were organized based on the progression of the implementation process.

### Lack of a needs assessment and engagement with key stakeholders

All participants thought that a needs assessment with practitioners and patients, described as a crucial element of healthcare implementation, was absent from CDMI. Participants also believed that some key players of project implementation were not sufficiently engaged prior to implementation, such as the nurses who were ultimately responsible for delivering the technology-based intervention. Participants suggested that performing a needs assessment, prior to the implementation would have facilitated staff buy-in through the identification of possible challenges and barriers. This would also increase awareness of possible facilitators.

*“…there being a needs assessment prior to the planning of the whole research, those needs could have been addressed and there would have been greater buy-in by the clinics… contacting the clinics saying that we would like to sit with them and create first of all, some sort of a questionnaire regarding needs assessment in order to assess the needs involved…” –Participant 1.*

Beyond a formal needs assessment, participants simply acknowledged that the opportunity for stakeholders to provide feedback and be heard was largely missing.

“We have to step back and make sure we've got the pathways and models of care really, fully engaged…making sure that we engage the key players in each of the family health teams. I think we thought we were reaching the right people but as often is the case, the people most impacted I don't think got enough say in it…” –Participant 4

### Lack of buy-in from medical staff

The lack of buy-in from frontline healthcare practitioners was identified by all participants as a factor leading to the unsuccessful implementation. Participants believed role clarity and expectations were unclear, and this negatively impacted buy-in. This challenge was augmented by ambiguity around the possible long-term benefits of the program to both patients and providers. The team-based approach to COPD care through smartphone messages was meant to reduce caseload burden in the long-term. However, participants felt this was poorly communicated to providers, and most participants felt the program increased workload.

“I think the primary care docs felt it was an add-on to their regular work. So did their staff. It wasn't clear as to who was going to be responsible for responding to the text messages. It wasn't clear how frequent the text messages would be”—Participant 3

Participants felt this could have been better communicated by first explaining how the CDMI program could complement their day-to-day responsibilities in the long term. To increase buy-in, participants felt that key components should have been better communicated, including implementation instructions, roles and responsibilities, along with the expected benefits. Participants noted a divide between the research team and the healthcare providers on the ground involved in implementation.

“I think that the folks that were involved with designing the project could see the big picture and could see how this could make things less work in the long run, in exchange for maybe more effort during the study period … the [medical] teams had voiced concern that they weren’t seeing value in proceeding with this [the CDMI project], and as a result they were not very invested in doing this kind of work because to them, it was just extra work.” –Participant 2

Despite the research team being interdisciplinary, some participants noted an apparent lack of awareness of how the program would interact with daily clinic work, resulting in unrealistic expectations and inadequate planning. One example given was around the lack of consideration for the workload of family physicians, who were tasked with recruitment, which lead to frustration and lack of support. The lack of incentive for participating in CDMI was discussed by participants as contributing to poor overall participation and recruitment.

“So for example the psychiatry and medicine folks I got the feeling that they thought ‘well, this should be easy for family docs to identify patients that could be for them easily recruited and to get the cell phones and everything should flow really smoothly’, yet the family docs weren’t really onboard and recruitment was slow. The staff were not buying into the text messaging thing and I don't think others really appreciated how difficult that was.” –Participant 3

### Inadequate patient engagement

Three interviewees attributed inadequate patient engagement to implementation failure. They highlighted that patients were not involved in CDMI’s planning or design, meaning patient perspectives and preferences were insufficiently incorporated.

“…it probably would have been useful to have a patient involved earlier. We might have learned that they weren't going to use these phones for example…” –Participant 4

Participants discussed challenges they felt patients had encountered such as paying for parking, working with technology, and having to conduct multiple tests. Despite trying to make study appointments around the same time as a patient’s regular visit, study appointments were often much longer in duration and required patients to complete extra paperwork. These added inconveniences were a deterrent to overall program momentum. In addition, participants felt technological support for platform’s roll-out for both providers and patients was difficult to access, leading to reduced engagement from patients and providers. The technology meant to improve care was perceived as a barrier for patients recruited and also hampered further recruitment efforts.

“… but the patients themselves had difficult using [the smartphone technology] for very practical reasons. Most of the folks with CHF and COPD are not young people, right?… So maybe if we limited the inclusion criteria to a younger batch, maybe the outcome would’ve been different.”” - Participant 2

“…people were sort of already using some of it and then we had some technical glitches too.” – Participant 4

### Contextual barriers

Participants acknowledged their context (an academic family health centre) was an important consideration both at the planning level and throughout implementation. An interesting paradox of the interdisciplinary nature of the research team came from the funding requirement for an interdisciplinary team. While touted as being a strength in the planning process, challenges arose with this diverse team during implementation since many of these researchers and practitioners had not regularly worked together prior to CDMI.

“… but I would have to say that the people involved on the [research team] didn't really know each other that well or really had a good understanding of how the other folks on the [research team] conducted their business or at least that was one factor that was kind of missing. I don't know how you correct that really because there was a group from psychiatry, there was a group from internal medicine, and there was family medicine, and then there were other people contributing to the study design and evaluation.” – Participant 3

This also brought an additional layer of complexity around evaluation of the programs with different disciplines valuing different metrics.

Lack of program resources posed obstacles to project implementation. This was apparent in the cell phones provided to clinics and patients.

“…the donation phones initially from [provider names] and they were old … and the buttons are miniscule.” – Participant 2

“Yeah, because most people I think especially in the [city] area probably had better [mobile] devices already and they weren't wanting to go back [to using older phones]…The telephone company did give us the phones free which was good, but patients didn’t really want to use the older phones.” – Participant 4

Staff turnover within the CDMI team was discussed as impacting both planning and implementation. During the planning and implementation stages of the project, several key project staff stepped away from their role either temporarily or permanently. Although their roles were filled, this unexpected turnover required additional time to integrate roles, and slowed the momentum.

“I think people were really affected when [Team Member A] was lost to us, although [Team Member B] came in, you know, just it's hard to keep the momentum in the same kind of direction.” – Participant 4

The CDMI program required clinic staff to be directly involved in program implementation. Thus, the research team did not have full control over the personnel involved in the project, which was acknowledged to a challenge for implementation.

“I mean we tried to mitigate that [the lack of control over personnel], right, by having the directors of the family health teams be part of the project. But at the end of the day you don’t have full control over personnel, and that’s reality, right? That’s what we learned from this project.” –Participant 2

## Discussion

The themes gathered from this study may seem intuitive and they have certainly been demonstrated as important in the literature, however they highlight the complexity and interconnectivity of these factors in practical application. They also demonstrate the need for a strong implementation plan to guide both program planning and implementation processes. Even when individual implementation barriers are accounted for, complex and interconnected barriers may still arise. This is especially true if the proper and continuous engagement of all key stakeholders is not done well. For our study, it was clear that having an interdisciplinary research team was necessary for enhancing the planning process; however, it was not sufficient for identifying all barriers and it may have hindered implementation success.

A poor theoretical basis for implementation guidance can make retrospective analysis of failed implementations difficult [16]. When implementation is guided by theory, it is more likely to succeed. The CDMI program was theoretically grounded during its planning stages [9], however, the implementation phase lacked theoretical guidance. Even though five of the six theoretical components proposed by Bodenheimer and colleagues [9] were considered in planning (buy-in by health care organizations, self-management support, structured practice teams for chronic care management, decision support and clinical information systems [9]), they lacked practical and contextual consideration for implementation.

With the exception of linkage with community resources, all other elements suggested by Bodenhiemer were incorporated into program planning. First, buy-in was achieved during program planning through an interdisciplinary team, with active participation from family physicians, and specialists in COPD, as well as psychiatry. Second, a central feature of CDMI was a navigator role to enhance patient self-management support and to support program implementation. This self-management support, although well intention, did not work well in practice largely due to insufficient consultation with providers and lack of patient engagement in planning. Third, structured practice teams were enabled through the proposed coordination of the navigator and primary care provider, with guidance from specialists, to share the responsibility of assessing and monitoring patients’ through the smartphone platform (including symptoms of depression, anxiety, adherence to medications and follow-up appointments). Fourth, decision support was incorporated through the navigator role who was meant to be available to support implementation and any technology trouble-shooting. Lastly, clinical information systems were a key design feature of the program; existing electronic medical records system were integrated with the programs text messaging to provide reminders to patients and feedback to providers. Linkage with community resources was not considered during program planning or implementation. Our study identified the several shortcomings in the application of the proposed constructs by Bodenheimer and colleagues [9]. First, the lack of patient engagement and provider consultation meant that the program was not aligned with the needs patients or requirements of providers. Second, while buy-in from organizational leads was obtained in planning, similar buy-in from front-line providers for implementation was not achieved. Third, decision support and clinical information systems were felt to duplicate existing services without clarity on expected outcomes for patients or providers. Lastly, interdisciplinary collaboration was, for the most part, only done at the planning level, and less so at the implementation and patient care level.

There are several implementation models that could have been used to support the success of CDMI such as CFIR or the PARiHS (Promoting Action on Research Implementation in Health Services) framework. PARiHS defines successful implementation as a function of three factors: evidence, context, and facilitation [17]. Adherence to this framework may have facilitated the necessary ‘pre-work’, such as conducting a proper needs assessment with patients, staff, and other stakeholders. Ecological theories of implementation such as the Active Implementation Framework [18] or Durlak and DuPre’s Ecological Framework [19] support a strong consideration for adaptability to factors like multiple stakeholders, complexities of health care systems, and the interconnectedness of variables [20]. Using a model, theory, or framework to practically support implementation with consideration for context is essential.

The benefits of interdisciplinary collaboration in research and program implementation are noted in the literature [21]. The CDMI research team was interdisciplinary, however, a lack of previous working relationships led to challenges in implementation. While interdisciplinary teams can support the likelihood of creating a successful intervention [22], equally as important is ensuring strong working relationships throughout implementation, building on previous successes (when available), and minimizing the risk to momentum if or when there is team member turnover.

There are several limitations of this study including the small sample size and the specific context in which the research took place. Having more study participants could have helped us develop a more complete picture of why this evidence-based program failed to successfully implement. Our findings are not meant to be generalized to other contexts, instead we believe our rich description of this unsuccessful implementation can provide lessons for other interventions – most notably, the importance of using theory to guide and support both planning and implementation, along with the importance of involving all stakeholders in both of these processes.

## Conclusion

In order to close critical gaps that currently persist in implementation science literature, reporting of implementation efforts including unsuccessful efforts is necessary. Describing unsuccessful interventions will support better understanding of how and why some evidence-based interventions do not succeed within a practical context. Understanding this can also develop our understanding of the complexity and interconnectivity of implementation factors within a specific context. These learnings can support improved methods for differentiating implementation-based failings and intervention-based failings, grow the literature surrounding failed implementation processes, and promote reflective learning from implementation efforts.

## Supplementary Information

**Additional file 1.**

**Additional file 2.**

## Data Availability

The datasets analyzed during the study are not publicly available due to the transcripts containing identifiable markers of healthcare providers but are available from the corresponding author on reasonable request after identifying details removed.

## Abbreviations

CDMI: Chronic Disease Management Initiative
CFIR: Consolidated Framework for Implementation Research
CHF: Congestive Heart Failure
COPD: Chronic Obstructive Pulmonary Disease
