# Supplementary material for: Lessons learned from inadequate implementation planning of team-based chronic disease management: implementation evaluation
Source: BMC Health Serv Res. 2021 Feb 12;21:134. doi: 10.1186/s12913-021-06100-4 (PMC7881538; doi:10.1186/s12913-021-06100-4)
Supplement: Supplementary file 1 — Additional file 1. [file 12913_2021_6100_MOESM1_ESM.docx]

**CDMI Team Interview Guide**

1. Can you describe the origins and development of the CDMI program?
2. How would you describe your involvement in CDMI?
3. Can you describe the decision-making process that occurred throughout the development and implementation of CDMI?
4. What were your perceptions of the program prior to implementation? Did those opinions change post implementation?
5. In your opinion, what are the strengths of CDMI?
6. Are there changes or improvements you feel should be made? If so, how would you accomplish these changes?
7. What are your opinions about including patients in program development and decision making?
8. Do you feel including patients in CDMI decision making would be beneficial? If so, how would you suggest doing so?
